# Designing consensus immunogens to break tolerance to self-antigens for cancer therapy

**DOI:** 10.18632/oncotarget.26275

**Published:** 2018-10-30

**Authors:** Elizabeth K. Duperret, Jian Yan, David B. Weiner

**Affiliations:** David B. Weiner: Vaccine & Immunotherapy Center, The Wistar Institute, Philadelphia, PA, USA

**Keywords:** tumor associated antigen, immune tolerance, DNA vaccine, fibroblast activation protein, Wilms tumor protein

Cancer vaccine immunotherapy has the promise to prime T cell responses in patients with T cell non-inflamed (‘cold’) tumors who do not respond well to immune checkpoint blockade. However, a major hurdle in developing efficacious cancer vaccines is designing immunogens with the capacity to break tolerance to tumor antigens. Because tumors derive from normal tissues, the targetable antigens that they express are often subject to both central and peripheral immune tolerance. This consists of deletion of self-reactive T cell clones in the thymus, and clonal inactivation or anergy of self-reactive clones in the periphery. While it was previously thought that the vast majority of self-reactive clones in the thymus are eliminated during development, it was recently shown that the frequency of peripheral foreign antigen specific and self-antigen specific CD8^+^ T cells is similar in healthy volunteers [1]. Interestingly, unlike the foreign antigen specific cells, these self-reactive CD8^+^ T cells are anergic upon peptide stimulation. This anergy can be overcome with a strong stimulus *in vitro*, suggesting that these cells could become potent killers with the proper stimulus *in vivo*. This is consistent with the observation that peripheral tumor-antigen specific T cells are detected in cancer patients, but are not potent enough to clear the patient’s tumor.

It has become apparent that immune tolerance can be broken in the context of autoimmune disease. A major trigger for the development of autoimmune disease is infection with viruses or bacteria. One mechanism for this is epitope mimicry, in which pathogens with sequences that have similarities to self-antigens provide a strong immune stimulus while triggering cross-reactivity to the self-epitope. MHC class II polymorphisms are associated with development of autoimmune disease, implicating this antigen presentation pathway in breaking tolerance.

Early vaccines targeting tumor antigens that relied on native antigen sequences were not potent in clinical studies. More sophisticated approaches employed xenogeneic antigens. The first description of this approach utilized a human tyrosinase protein immunogen to break tolerance in mice, inducing both anti-tumor immunity as well as coat de-pigmentation [2]. Early clinical studies using mouse immunogens in humans have also shown some immune responses in melanoma patients [3]. A proposed mechanism for xenogeneic immunogens is the introduction of heteroclictic epitopes [4]. However, this mechanism is likely to be highly HLA specific, and may vary among individuals and among antigens. Another approach for breaking tolerance has been to introduce mutations randomly using error-prone polymerase chain reaction [5]. While this approach was effective in generating anti-tumor immunity, it was rather inefficient with only one immunogenic protein per 239 mutations [5]. Rational design of immunogens has also been explored, with selected mutations that can improve processing or MHC presentation of epitopes [6]. However, this approach is MHC specific and will require personalized vaccine design.

In our laboratory, we have advanced the concept of xenogeneic and randomly mutated vaccines using a synthetic consensus (SynCon) approach to intelligently engineer diversity, allowing for retention of the structure of the native antigen as well as breaking of tolerance in diverse individuals. Much of this concept originated from the study of variable pathogen vaccines, in which we identified optimal consensus immunogens that could generate both robust antibody and T cell responses to diverse strains.

**Figure 1:**
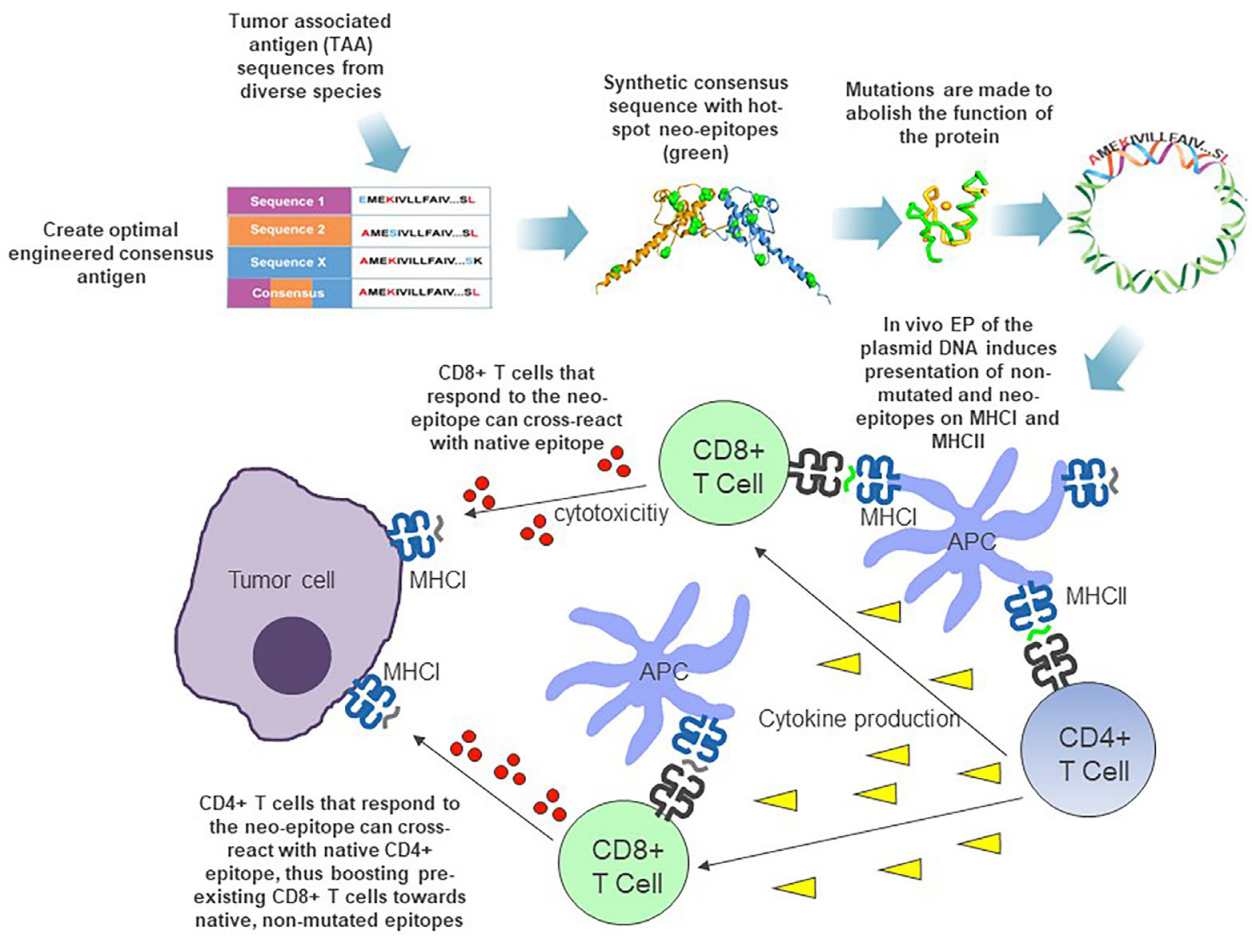
Synthetic consensus immunogen design and mechanism of action

For adapting this SynCon approach for cancer antigens, we engineer a consensus sequence generated from diverse species that retains a pre-defined homology (of approximately 95%) to the native antigen. In this consensus sequence, we employ broad spacing of neo-epitopes (1 per 20 amino acids) to ensure a high ratio of MHC class II to MHC class I neo-epitopes. Conservation of major structural features of the antigen is important. This consensus focus retains structure of the antigen, encouraging proper processing and potential development of specific antibodies, if relevant. In addition, biological functions for these cancer antigens are ablated to avoid potential oncogenic function, (Figure 1) and the incorporation of novel leader sequences can change the intracellular location and/or secretion of the antigen, thus influencing the presentation of the designed immunogen.

We have reported that this approach is superior to the native sequences for breaking tolerance and driving CD8^+^ immunity to two important cancer antigens: WT1 and FAP [7, 8]. For the WT1 antigen, we were unable to detect significant immune responses to the native mouse immunogen; however, the SynCon antigen generated robust immune responses and breaking of tolerance [8]. We performed epitope mapping, where we observed that auto-immune responses were induced to diverse epitopes, including peptides that were minimally altered as well as self-peptides that were not mutated (Figure 1). This indicates that the consensus immunogen was able to induce epitope spreading to non-mutated epitopes, potentially through MHC class II antigen presentation.

We extended these analyses to diverse strains of mice using the SynCon FAP immunogen [7]. This immunogen generated robust immunity and broke tolerance, whereas the native antigen was much poorer immune performer in outbred mice [7]. As FAP is a surface antigen, antibody responses may be important for anti-tumor immunity. In Th2-driven Balb/c mice, the native FAP immunogen was unable to generate antibody titers, while the SynCon FAP immunogen generated robust titers in the majority of mice immunized [7].

Taken together, these examples highlight that SynCon immunogens may have unique advantages for generation of consistent, robust self-antigen immunity in diverse individuals. Clinical studies utilizing this approach are ongoing for the tumor antigens TERT, WT1 and PSMA (NCT 02960594, NCT03502785, NCT03491683, NCT02514213).
